# Augmented Reality in Pediatric Septic Shock Simulation: Randomized Controlled Feasibility Trial

**DOI:** 10.2196/29899

**Published:** 2021-10-06

**Authors:** Regina L Toto, Ethan S Vorel, Khoon-Yen E Tay, Grace L Good, Jesse M Berdinka, Adam Peled, Marion Leary, Todd P Chang, Anna K Weiss, Frances B Balamuth

**Affiliations:** 1 Division of Emergency Medicine Department of Pediatrics The Children's Hospital of Philadelphia Philadelphia, PA United States; 2 BrickSimple, LLC Doylestown, PA United States; 3 University of Pennsylvania School of Nursing Philadelphia, PA United States; 4 Division of Emergency Medicine & Transport Children's Hospital Los Angeles Los Angeles, CA United States

**Keywords:** augmented reality, simulation, septic shock, children, pediatrics, simulation-based education, application, fluid administration

## Abstract

**Background:**

Septic shock is a low-frequency but high-stakes condition in children requiring prompt resuscitation, which makes it an important target for simulation-based education.

**Objective:**

In this study, we aimed to design and implement an augmented reality app (PediSepsisAR) for septic shock simulation, test the feasibility of measuring the timing and volume of fluid administration during septic shock simulation with and without PediSepsisAR, and describe PediSepsisAR as an educational tool. We hypothesized that we could feasibly measure our desired data during the simulation in 90% of the participants in each group. With regard to using PediSepsisAR as an educational tool, we hypothesized that the PediSepsisAR group would report that it enhanced their awareness of simulated patient blood flow and would more rapidly verbalize recognition of abnormal patient status and desired management steps.

**Methods:**

We performed a randomized controlled feasibility trial with a convenience sample of pediatric care providers at a large tertiary care pediatric center. Participants completed a prestudy questionnaire and were randomized to either the PediSepsisAR or control (traditional simulation) arms. We measured the participants’ time to administer 20, 40, and 60 cc/kg of intravenous fluids during a septic shock simulation using each modality. In addition, facilitators timed how long participants took to verbalize they had recognized tachycardia, hypotension, or septic shock and desired to initiate the sepsis pathway and administer antibiotics. Participants in the PediSepsisAR arm completed a poststudy questionnaire. We analyzed data using descriptive statistics and a Wilcoxon rank-sum test to compare the median time with event variables between groups.

**Results:**

We enrolled 50 participants (n=25 in each arm). The timing and volume of fluid administration were captured in all the participants in each group. There was no statistically significant difference regarding time to administration of intravenous fluids between the two groups. Similarly, there was no statistically significant difference between the groups regarding time to verbalized recognition of patient status or desired management steps. Most participants in the PediSepsisAR group reported that PediSepsisAR enhanced their awareness of the patient’s perfusion.

**Conclusions:**

We developed an augmented reality app for use in pediatric septic shock simulations and demonstrated the feasibility of measuring the volume and timing of fluid administration during simulation using this modality. In addition, our findings suggest that PediSepsisAR may enhance participants’ awareness of abnormal perfusion.

## Introduction

### Background

Augmented reality (AR) and virtual reality (VR) have been increasingly explored as tools for innovation in medical education in both medical and surgical subspecialties [1-3]. VR refers most broadly to the digital representation of an immersive world, whether novel or realistic [1,4]. In contrast, AR projects a digital overlay onto the physical environment, emphasizing task performance in the real world that is *augmented* by virtual elements [2]. AR and VR may be experienced through head-mounted displays using mobile devices or computers. In recent years, AR- and VR-based activities have become increasingly easy to scale and distribute widely [4,5].

AR and VR have been used most frequently for surgical training [6-8], anatomical study [9-11], and cardiopulmonary resuscitation (CPR) training [12-14]. To date, there is a relative dearth of studies exploring the incorporation of AR into medical resuscitation scenarios [2,15,16]. Studies evaluating AR enhancement of simulated procedural training indicate that learners respond favorably to its use [17-19]. In a recent medical simulation study, participants recognized shock and clinical decompensation significantly sooner when using AR. These findings suggest the potential of AR to improve clinical care at the bedside [17].

Simulation has been shown to enhance retention of resuscitation skills [20]; pediatric care providers particularly benefit from simulation-based resuscitation exercises, as real-life resuscitation events are rare in children [21,22]. However, even high-fidelity simulations are limited in their capacity to replicate real-life scenarios. These challenges are highlighted in the case of sepsis, a potentially life-threatening response to infection. In children with potential sepsis, clinicians need to make rapid clinical decisions [23], and these decisions are often based on physical examination findings that are difficult to simulate on a mannequin. Time to recognize septic shock and administrate antibiotics and intravenous fluids are all linked to improved outcomes in pediatric patients with septic shock [24]. For this reason, many hospitals have implemented recognition systems and care pathways to ensure rapid, standardized, and high-quality care for children in septic shock; our institution’s sepsis pathway is one example. By providing a means of visualizing perfusion during simulation, AR can potentially enhance simulation participants’ abilities to make timely and realistic management decisions about patients in septic shock.

### Objective

In this study, we aimed to create an AR representation of impaired perfusion (PediSepsisAR) and incorporate this app into a pediatric septic shock simulation. Our primary aim was to determine the feasibility of collecting data on the timing and volume of fluid administrated during septic shock simulation with and without the use of PediSepsisAR. We hypothesized that we would be able to measure the timing and volume of fluid administration in 90% of the participants. A second exploratory aim was to describe PediSepsisAR as an educational tool in septic shock simulation. Specifically, we aimed to compare control and PediSepsisAR participants’ timing of stated recognition of shock and desired management steps and elicit participant attitudes toward the experience of using PediSepsisAR during septic shock simulation. We hypothesized that PediSepsisAR participants would sooner state shock recognition and express desired management steps—administering antibiotics and initiating the sepsis pathway. In addition, we hypothesized that participants would report that PediSepsisAR enhanced their awareness of the simulated patient’s perfusion; therefore, the use of the app would have made them want to administer fluids more quickly.

## Methods

### Theoretical Framework

In designing and studying PediSepsisAR, we considered two main underlying principles: real-time feedback and gamification. Studies have demonstrated improved CPR performance in both simulated and real-life resuscitations using CPR feedback devices [25,26]. By adding PediSepsisAR to a traditional septic shock simulation, we hoped to convey a visual representation of poor perfusion that participants could monitor for improvements as they administered fluids. Real-time feedback is closely tied to the gamification of simulation exercises. Specifically, Rutledge et al [27] define gamification as the addition of a design element to an existing learning activity to facilitate achieving the activity’s goals. In this manner, PediSepsisAR can be viewed as an element applied to septic shock simulation that provides a dynamic visual representation of the simulated patient’s circulation. PediSepsisAR provides ongoing real-time feedback on the participants’ progress toward the goal of fluid resuscitating the simulated patient; thus, it facilitates the achievement of the goal and gamifies the simulation.

### Study Setting

This study was conducted in the pediatric emergency department of a large tertiary care academic children’s hospital from October 10 to November 12, 2020.

### Study Participants

We enrolled a convenience sample of 50 participants, including pediatric residents, pediatric emergency medicine (PEM) fellows, PEM attending physicians, nurse practitioners, and pediatric nurses, who regularly practice at the pediatric emergency department in the study hospital. Participants were recruited via a combination of email and in-person communication. Written informed consent was obtained from all participants.

### Study Design

This study consisted of a nonblinded, randomized, controlled trial design.

### Prototype Design

**Figure 1:**
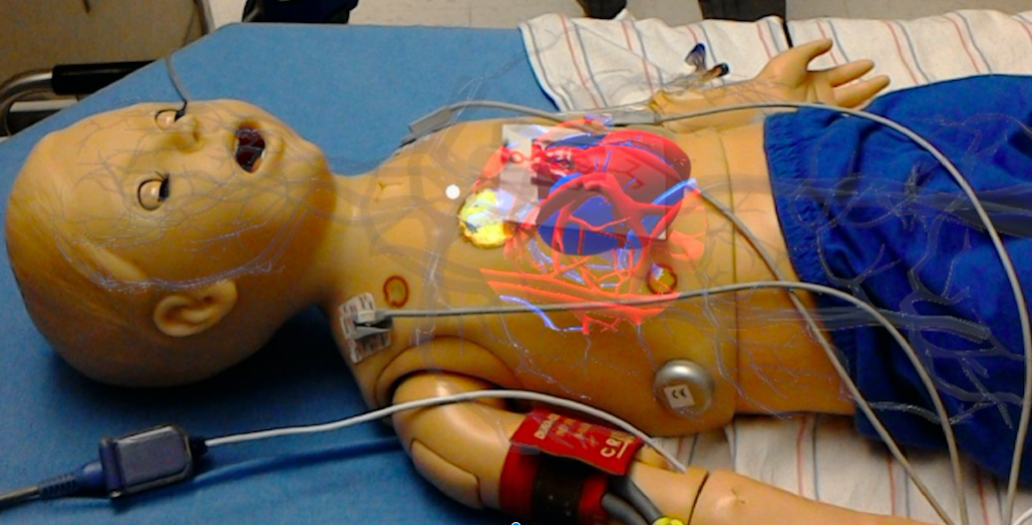
PediSepsisAR overlaid on the simulation mannequin, as visualized through the HoloLens.

Through a partnership with BrickSimple, LLC, a Philadelphia-based software company, we adapted an existing prototype, CPReality [28,29], to create PediSepsisAR. CPReality integrates with the first-generation Microsoft HoloLens headset and allows for the depiction of a digital model of the circulatory system that can be overlaid on a simulation mannequin. CPReality integrates data from actual CPR performance on a mannequin to create a digital image of the *patient’s* circulation, which allows participants to visualize the effect of their chest compressions on perfusion during CPR. Our aim in adapting this prototype was to enable our participants to visualize a model of impaired perfusion during fluid resuscitation in a simulated patient with septic shock. Initially, the vessels most proximal to the heart are illuminated, demonstrating that peripheral perfusion is limited (Figure 1); after 20, 40, and 60 cc/kg of fluid boluses are administered, perfusion spreads distally until it ultimately reaches the brain and most peripheral tissues. A demonstration of the circulation expansion is shown in Multimedia Appendix 1.

We worked with BrickSimple to enable PediSepsisAR to interact with a potentiometer embedded in an intravenous fluid syringe. A potentiometer is a variable resistor that linearly restricts low amounts of electricity that can then be translated into an electrical signal. A linear slide potentiometer was chosen specifically because the sliding effect fits well with the mechanical action of pushing a plunger. Team members fit the potentiometer into a 60-cc syringe and then attached the moving part of the device (Wiper) to the syringe plunger so that it would move in a relative fashion depending on how far the plunger was depressed. The position of the plunger could be measured against time to measure the rate of fluid bolus administration.

**Figure 2:**
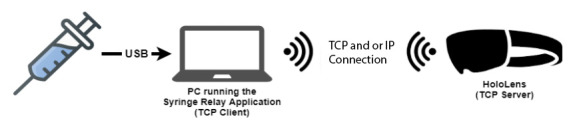
The sequence of communication between the potentiometer and HoloLens. Note that the potentiometer is embedded within the syringe displayed at the left. TCP: transmission control protocol.

Once the potentiometer was developed, it was necessary to ensure its ability to communicate with the HoloLens. This was achieved via a wireless connection. More specifically, the potentiometer is connected via USB to a computer running a custom app called Syringe Relay. The HoloLens app that provides the visuals also spins up a transmission control protocol or IP server. The Syringe Relay then connects to the transmission control protocol or IP server and uses it to send data to the HoloLens. The communication sequence is as follows: the potentiometer sends data to the Syringe Relay via the USB; the Syringe Relay app then processes and forwards the data to the HoloLens via a wireless connection. This sequence is shown in Figure 2.

For the flow of communication to work properly, both the HoloLens and computer must be on the same wireless internet network. When launched, the PediSepsisAR app on the HoloLens continually scans for new connections. The Syringe Relay app is then configured with the current IP address of the HoloLens and can thereby connect to it and send data to it.

All data processing occurred within the Syringe Relay app. First, the app maps the raw potentiometer values to the current milliliter value on the syringe. When the plunger is depressed, it adds the delta to a running total of how many milliliters are infused. The app then converts the total milliliters infused to total mL/kg and sends that value to the HoloLens. The HoloLens then updates the visual display of the perfusion accordingly.

To our knowledge, this is the first study using a potentiometer to measure real-time intravenous fluid administration during simulation.

### Study Procedures

**Figure 3:**
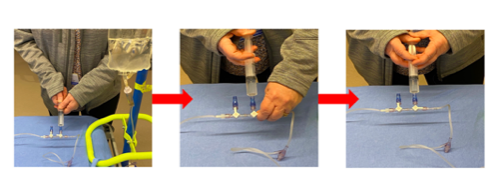
Push-pull technique for fluid administration. Fluid is manually pulled from the bag reservoir and then pushed into the patient.

All enrolled participants (n=50) completed an electronic prestudy questionnaire. All questionnaire data were collected and managed using REDCap (Research Electronic Data Capture) tools hosted at our institution [30,31]. The prestudy questionnaire was created de novo in conjunction with a coauthor AKW who has expertise in survey question development and is included as a reference in Multimedia Appendix 2. This questionnaire elicited participants’ demographic data and their report of previous experience with push-pull fluid administration, simulated resuscitations in general, and simulated septic shock. After completing the prestudy questionnaire, each participant opened an envelope containing a number; those participants whose envelope contained even numbers were randomized to the PediSepsisAR group, and those whose envelopes contained odd numbers were randomized to the control group. All participants had the opportunity to practice administering fluids using the push-pull technique (Figure 3) before the simulation exercise. Those randomized to PediSepsisAR then received a short orientation on the HoloLens, which reviewed the proper fit of the HoloLens and provided an example of the visual representation of circulation they would see through the HoloLens.

During the consent procedure, participants were informed that the investigators would evaluate their administration of fluids to a simulated patient with septic shock. They were invited to verbalize their assessment of the patient and the management steps they might take in addition to intravenous fluid administration. Before beginning the exercise, all participants received a standardized, short description of the clinical scenario: a previously healthy male aged 15 months presenting with 2 days of fever and 1 day of decreased oral intake and decreased responsiveness. Facilitators gave no further prompts during the simulation. Those randomized to the PediSepsisAR group wore the HoloLens during the simulation; those randomized to the control group completed the simulation without the use of AR. We used the Pediatric HAL (Gaumard) mannequin for all simulations. We measured all participants’ fluid administration using the potentiometer embedded in the 60-cc syringe used to push-pull fluids and recorded the time to administer 20, 40, and 60 cc/kg total administered fluids for the patient’s stated weight. Participants were not directed to administer a specific amount of fluids, and those who chose to stop administering fluids at any point in the simulation were allowed to do so. In addition, the facilitator marked the time that the participant verbalized the following elements of sepsis recognition and treatment: tachycardia or hypotension, sepsis, shock, septic shock, initiation of the sepsis pathway, and intent to administer antibiotics.

Following the simulation exercise, those in the PediSepsisAR group completed an electronic poststudy questionnaire through REDCap asking their opinions about how PediSepsisAR did or did not affect their simulation experience. The poststudy questionnaire was created de novo. Participants had the opportunity to elaborate in a free-text form regarding their answers to the survey questions. The poststudy questionnaire is included in Multimedia Appendix 3. All participants received a US $5 coffee shop gift card following their participation.

This study was approved by the institutional review board of the Children’s Hospital of Philadelphia. We used Stata 16.0 (StataCorp) for statistical analysis. We assessed the study population using descriptive statistics and compared the median time with the administration of 20, 40, and 60 cc/kg of fluids between each group using the Wilcoxon rank-sum test.

### Feasibility Endpoints

We defined the following endpoints a priori as reaching our feasibility goal: (1) We hypothesized that we could feasibly create PediSepsisAR and enable it to interact with a fluid administration system and (2) we hypothesized that we could measure the timing and volume of fluid administration using the potentiometer in at least 90% of the participants. In addition, we hypothesized that for the PediSepsisAR participants, the potentiometer and AR interactivity would function as intended at least 90% of the time.

## Results

### Overview

We enrolled a total of 50 interprofessional participants, including 6 nurses, 11 nurse practitioners, and 33 physicians at different levels of training: 10 pediatric residents, 13 PEM attendings, and 10 PEM fellows. In total, 25 participants were randomized to the control group, and 25 participants were randomized to the PediSepsisAR group; all disciplines were represented in both the PediSepsisAR and control groups. The demographic characteristics of the study population are shown in Table 1. In the presimulation questionnaire, most participants reported that they had given push-pull intravenous fluids before (21/25, 84% in both groups). Similarly, most participants reported previous experience with septic shock simulation (23/25, 92% in the PediSepsisAR group and 19/25, 76% in the control group). A small minority of participants in either group had previous experience with AR (0/25, 0% in the PediSepsisAR group and 2/25, 8% in the control group).

**Table 1:** Demographic data for study participants (N=50).

Participants enrolled		PediSepsisAR (n=25), n (%)	Control (n=25), n (%)
**Study population**			
	Nurse	4 (16)	2 (8)
	Advanced practice provider	5 (20)	6 (24)
	Fellow	6 (24)	5 (20)
	Resident	5 (20)	6 (24)
	Attending	5 (20)	6 (24)
**Clinical experience (years)**			
	1-5	6 (24)	3 (12)
	6-10	8 (32)	8 (32)
	>10	11 (44)	14 (56)
**Ever administered push-pull IVF^a^**			
	Yes	21 (84)	21 (84)
	No	4 (16)	4 (16)
**Times administered push-pull IVF in the past year**			
	None	12 (48)	10 (40)
	1-5	13 (52)	13 (52)
	>5	0 (0)	2 (8)
**Previous simulation experience**			
	Yes	23 (92)	25 (100)
	No	2 (8)	0 (0)
**Septic shock simulation experience**			
	Yes	23 (92)	19 (76)
	No	2 (8)	6 (24)
**Augmented reality experience**			
	Yes	0 (0)	2 (8)
	No	25 (100)	23 (92)

^a^IVF: intravenous fluid.

### Fluid Administration and Verbalization

We measured and recorded the timing of completed administration of 20, 40, and 60 cc/kg of fluids in all participants. In total, 16% (4/25) participants in the PediSepsisAR group experienced the inability to visualize PediSepsisAR initially through the HoloLens; in each instance, the study staff closed and reopened PediSepsisAR on the device, and participants were able to visualize PediSepsisAR after that. All 4 participants subsequently completed the simulation exercise uninterrupted. Participants in the PediSepsisAR group were slightly more likely than those in the traditional group to administer 60 cc/kg of fluid (relative risk 1.2, 95% CI 1.03-1.41). For participants who elected not to give the third bolus, the 60 cc/kg time point was not included. The median time to administration of 20 cc/kg was 117 seconds (IQR 93-154) for the PediSepsisAR group and 134 seconds (IQR 98-161) for the control group (*P*=.68). For 40 cc/kg, the median time to administration was 265 seconds (IQR 229-363) for the PediSepsisAR group and 284 seconds (IQR 250-350) for the control group (*P*=.51). Finally, the median time to administration of 60 cc/kg was 419 seconds (IQR 377-536) for the PediSepsisAR group and 468 seconds (IQR 392-524) for the control group (*P*=.47). These data are presented in Table 2.

**Table 2:** Time to fluid bolus in seconds displayed as median (IQR), PediSepsisAR versus control groups.

Fluid bolus	PediSepsisAR (n=25)			Control (n=25)			*P* value
	Time (seconds), median (IQR)	Participants, n (%)	Time (seconds), median (IQR)		Participants, n (%)		
20 cc/kg	117 (93-154)	25 (100)	134 (98-161)		25 (100)	.68	
40 cc/kg	265 (229-363)	25 (100)	284 (250-350)		25 (100)	.51	
60 cc/kg	419 (377-536)	25 (100)	468 (392-524)		21 (84)	.47	

In addition to recording the volume and timing of fluid administration, we recorded the points at which participants verbalized recognizing the patient’s condition and expressed the desire to take certain management steps; 92% (23/25) of participants in the PediSepsisAR group and 100% (25/25) of those in the control group verbalized recognizing tachycardia or hypotension. The median time to verbalized recognition was 26 seconds (IQR 6-43) for the PediSepsisAR group and 39 seconds (IQR 10-95) for the control group (*P*=.20). Regarding the desire to initiate the sepsis pathway, 32% (16/50) of participants verbalized that they would like to start the pathway; this included 20% (5/25) of participants in the PediSepsisAR group and 44% (11/25) of participants in the control group. The median time to verbalize the desire to initiate the sepsis pathway was 96 seconds (IQR 90-133) for the PediSepsisAR group and 136 seconds (IQR 40.5-421) for the control group (*P*=.67). The data for *stated shock*, *septic shock*, or *sepsis* are as follows: 56% (14/25) of participants in the PediSepsisAR group verbalized one or more of these terms versus 68% (17/25) in the control group. The median time to verbalized recognition of *shock*, *septic shock*, or *sepsis* was 66 seconds (IQR 34-94) for the PediSepsisAR group and 87 seconds (IQR 23.5-192) for the control group (*P*=.84). In both, the PediSepsisAR and control groups, 84% (21/25) of participants requested antibiotics. Of those who requested antibiotics, the median time to request was 81 seconds (IQR 53-167) for the PediSepsisAR group and 165 seconds (IQR 28-198) for the control group (*P*=.96). These data are presented in Table 3.

**Table 3:** Time to verbalize specific patient status items and management steps in seconds displayed as median (IQR), PediSepsisAR group versus control group.

Verbalization	PediSepsisAR (n=25)		Control (n=25)		*P* value
	Time (seconds) taken to verbalize, median (IQR)	Participants who verbalized, n (%)	Time (seconds) taken to verbalize, median (IQR)	Participants who verbalized, n (%)	
Tachycardia ± hypotension	26 (6-143)	23 (92)	39 (10-95)	25 (100)	.20
Sepsis pathway	96 (90-133)	5 (20)	136 (40.5-421)	11 (44)	.67
Shock, septic shock, or sepsis	66 (34-94)	14 (66)	87 (23.5-192)	17 (68)	.84
Antibiotics	81 (53-167)	21 (84)	165 (28-198)	21 (84)	.96

In the poststudy questionnaire, most participants in the PediSepsisAR group (23/25, 92%) reported that the addition of AR enhanced their awareness of the patient’s blood flow. When asked whether PediSepsisAR made them want to push fluids faster, 56% (14/25) of the PediSepsisAR participants answered that it did. Many participants remarked that PediSepsisAR was distracting (8/25, 32%). In free-text comments, some participants reported that the digital visualization of circulation allowed them to appreciate the patient’s fluid responsiveness in a new way. Some participants commented on the limited field of vision through HoloLens.

## Discussion

### Principal Findings

In this study, we aimed to design an AR app that could be integrated into a pediatric septic shock simulation and demonstrate the feasibility of its use. Our results demonstrate that it is feasible to record the timing and volume of fluid administration during septic shock simulation both with and without the addition of AR. We hypothesized that we would be able to capture such data in at least 90% of participants in each group and ultimately captured the desired data in all participants.

Our study was not powered to detect significant differences in times of fluid administration between the control and PediSepsisAR groups. We observed that the PediSepsisAR group had a shorter time to fluid bolus administration than the control group. More than half of the participants in the PediSepsisAR group reported that PediSepsisAR made them want to push fluids faster, suggesting that perhaps the components of simulation (provided history, vital signs, and reported delayed capillary refill) alone may encourage rapid fluid administration even without an AR component. Of note, participants in the control group were slightly less likely to administer 60 cc/kg than participants in the PediSepsisAR group. The underlying reason for this observation remains unclear. The changes in simulated patient vital signs with each fluid bolus (20, 40, and 60 cc/kg) were the same for both groups; PediSepsisAR participants showed incompletely improved circulation at 40 cc/kg, whereas the control group participants did not. This suggests that perhaps the additional visualization of perfusion provided through PediSepsisAR informed participants’ awareness of the patient’s status in a way that vital signs alone could not. The question of whether visualizing a digital model of perfusion has an impact on the administration of intravenous fluids during simulation remains and can be explored in future studies.

Our secondary exploratory aim was to describe PediSepsisAR as an educational tool. We explored the educational value of PediSepsisAR in two ways: through comparison of verbalized recognition of patient status and desired management steps in both groups during the simulation exercise and poststudy questionnaires completed by the PediSepsisAR group. We did not find any significant differences in median times to verbalizations of key patient status recognition or management steps, as our study was not powered to detect such a difference. We observed that the time to verbalization of both patient status (abnormal vital signs and shock or septic shock) and requested sepsis pathway initiation and antibiotics were shorter in the PediSepsisAR group than in the control group. This finding suggests that PediSepsisAR may aid the recognition of abnormal fluid status and, in doing so, allow participants to plan management steps more efficiently. Future studies are needed to explore whether the addition of AR impacts the assessment or delivery of care to simulated patients.

The poststudy questionnaire results revealed that most participants perceived that PediSepsisAR enhanced their awareness of the simulated patient’s perfusion. Traditional high-fidelity simulation enables participants to palpate pulses and visualize capillary refill [32], allowing them to assess the simulated patient’s perfusion. Nevertheless, capillary refill serves as a surrogate marker of perfusion in both simulated and human patients and interprovider assessment of capillary refill lacks reliability [33]. The novel representation of perfusion provided through PediSepsisAR provides visual information of the simulated patient’s condition that participants would otherwise not receive. We connect this to our observation that PediSepsisAR participants had overall shorter times to fluid bolus administration, suggesting that AR representation of simulated patient perfusion may have affected the speed of fluid administration.

A key balancing metric from our poststudy questionnaire is that more than 32% (8/25) of the participants in the PediSepsisAR group found the app distracting. Some participants reported in free-text comments that they had difficulty toggling between viewing digital media through the HoloLens and the physical monitor displaying vital signs, which was positioned beside the mannequin. Most of our participants had engaged in simulated septic shock scenarios before but had no previous experience with AR. It is possible that their lack of familiarity with the experience of visualizing digital media in the physical world contributed to their assessment of PediSepsisAR as distracting. Yet, it is important to consider not only the potential benefits but also the disadvantages of incorporating additional technology into an already effective educational practice such as high-fidelity simulation. It is also possible that certain learners would benefit more from AR-based educational strategies than others, which could be explored in future studies.

### Limitations

This study has several important limitations. First, we address the limitations of assessing the feasibility of collecting data on the timing and volume of fluid administration with and without PediSepsisAR. Our fluid measurement system, though novel, also has limitations. In this feasibility study, we measured the timing and volume of fluid administration using a potentiometer embedded in a syringe. Because these measurements are based on the movement of the potentiometer and not on the actual volume of fluid administered, the air within the syringe could affect the measurements. It is possible that the fluid administration times for some participants were falsely shortened as a result.

Regarding generalizability, the cost of AR app development and head-mounted displays such as the HoloLens may hinder replicating the design of an AR model of circulation and its integration into a simulation. The potentiometer that we used to measure the timing and volume of fluid administration required engineering expertise. In addition, learning how to use and troubleshoot PediSepsisAR required time investment and technological support from our colleagues at BrickSimple. Therefore, lack of protected time for educational innovation, staffing constraints, and lack of funding for adequate support can all be potential roadblocks to replicate our study in other environments.

Second, we address the limitations of evaluating PediSepsisAR as an educational tool. The limited field of view provided by the first-generation HoloLens used in this study could have negatively affected participants’ experiences during the simulation. In particular, suspension of disbelief becomes more challenging as participants experience technology glitches during simulated exercise. Future studies displaying AR app through the HoloLens may benefit from using the second-generation HoloLens or the HP Mixed Reality headset, which have larger fields of view.

PediSepsisAR provides a simple representation of the complex pathophysiology of septic shock. With our available funding, we could integrate the CPReality prototype with the fluid administration system, but we were unable to make the perfusion model more realistic. There are many potential opportunities to make PediSepsisAR a more accurate representation of septic shock. These include, but are not limited to, adding a digital model of impaired capillary refill or skin findings such as mottling or petechiae. A more nuanced model could be configured to reflect improved or worsened perfusion depending on the type of shock (septic vs cardiogenic) and the participants’ chosen interventions. Specifically, an AR model of cardiogenic shock could depict impaired distal perfusion with a weakly pumping heart that pumps blood less effectively after the administration of fluids. Visualizing interactive AR models of both cardiogenic and septic shock alongside one another may allow participants to better recognize their differences and distinguish between the two during a simulated case of undifferentiated shock. Future studies should investigate AR models of septic and cardiogenic shock as educational tools to enhance simulation-based recognition of these pathologic states.

### Conclusions

In conclusion, this randomized study demonstrated that it is feasible to measure the time to fluid bolus administration during pediatric septic shock simulation. Preliminary findings from our exploratory aims suggest that incorporating PediSepsisAR into septic shock simulation may enhance participants’ awareness of the simulated patient’s perfusion. Future studies can explore whether the addition of AR affects participant performance, including fluid administration, in septic shock simulation.

## Appendix

Multimedia Appendix 1 Video 1 demonstrates the incremental expansion of digital circulation as participants administer fluid boluses during the simulation. The first expansion occurred after the administration of 20 cc/kg, the second at 40 cc/kg, and the final expansion at 60 cc/kg.

Multimedia Appendix 2 Prestudy questionnaire administered to all participants.

Multimedia Appendix 3 Poststudy questionnaire administered to participants in the PediSepsisAR group.

## Abbreviations

AR: augmented reality
CPR: cardiopulmonary resuscitation
PEM: pediatric emergency medicine
REDCap: Research Electronic Data Capture
VR: virtual reality
