# Supplementary material for: Augmented Reality in Pediatric Septic Shock Simulation: Randomized Controlled Feasibility Trial
Source: JMIR Med Educ. 2021 Oct 6;7(4):e29899. doi: 10.2196/29899 (PMC8529461; doi:10.2196/29899)
Supplement: Multimedia Appendix 2 [file mededu_v7i4e29899_app2.docx]

Please create a unique identifier.

This will be the last two letters of your last name followed by the numerical date of your birthday. For example, Regina Toto born on January 22 would be TO22.

­­­­­­­

1. Have you ever personally administered push-pull IV fluids to a real or simulated patient?

- Yes
- No

1. How many times in the past year have you personally administered push-pull IV fluids to a real or simulated patient?

- 0
- 1-5
- More than 5

Please select your role.

- Nurse
- Advanced Practice Provider (Nurse Practitioner or Physician Assistant)
- Physician- fellow
- Physician- resident
- Physician- attending

How many years of clinical experience, including medical / nursing school and training, do you have?

- 1-5
- 6-10
- More than 10

Have you ever participated in a simulated resuscitation (either medical or trauma) before?

- Yes
- No

If yes, have you ever participated in a simulated case of septic shock before?

- Yes
- No

Do you have any experience with augmented reality in the clinical environment?

- Yes
- No
