# Supplementary material for: Augmented Reality in Pediatric Septic Shock Simulation: Randomized Controlled Feasibility Trial
Source: JMIR Med Educ. 2021 Oct 6;7(4):e29899. doi: 10.2196/29899 (PMC8529461; doi:10.2196/29899)
Supplement: Multimedia Appendix 3 [file mededu_v7i4e29899_app3.docx]

Please enter your unique identifier.

1. Was this your first experience with augmented reality in simulation?

- Yes
- No

1. Did the addition of augmented reality enhance your awareness of the patient’s blood flow?

- Yes
- No

*Please describe. (either answer)*

1. Did you find the addition of augmented reality to the simulation to be distracting?

- Yes
- No

1. Did visualizing the simulated patient’s blood flow make you want to push fluids faster?

- Yes
- No

*Please describe. (either answer)*

1. Please provide any reactions or feedback to the augmented reality application you just experienced. *(free text)*
